# Endoscopic stricturoplasty for pyloric stenosis refractory to endoscopic balloon dilation and lumen apposing metal stenting

**DOI:** 10.1055/a-2749-3337

**Published:** 2026-01-08

**Authors:** Jonathan Rozenberg, Rohit Kumar, William F. Abel, Joel Joseph, Vivek Kesar, Patrick I. Okolo, Varun Kesar

**Affiliations:** 1 Department of Internal Medicine, Virginia Tech Carilion, Roanoke, Virginia, United States; 2 1757 Virginia Tech Carilion School of Medicine, Roanoke, Virginia, United States; 3 Department of Internal Medicine, Division of Gastroenterology, Virginia Tech Carilion, Roanoke, Virginia, United States; 4 Consultants in Gastroenterology, West Columbia, West Columbia, South Carolina, United States; 5 22161 Department of Internal Medicine, Division of Gastroenterology, Stony Brook University Hospital, Stony Brook, New York, United States

**Fig. 1:**
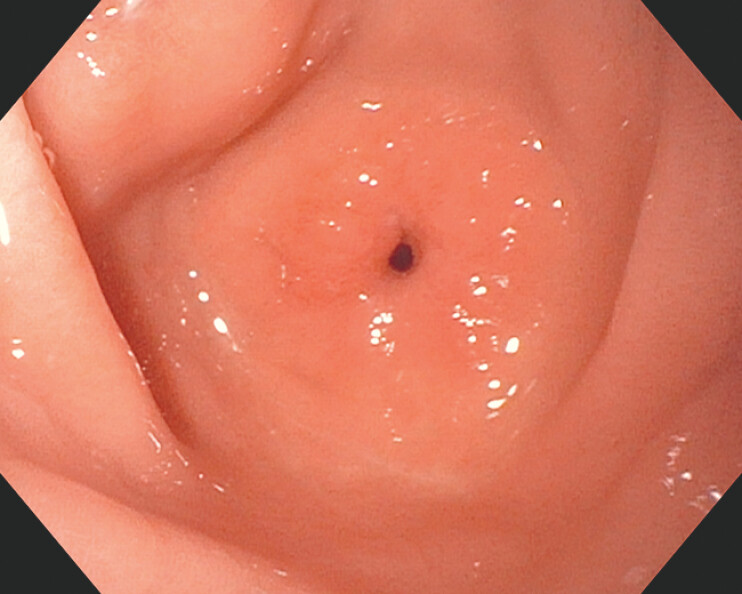
Abb. An endoscopic image of a tight stricture in the distal antrum with associated pyloric stenosis.

**Fig. 2:**
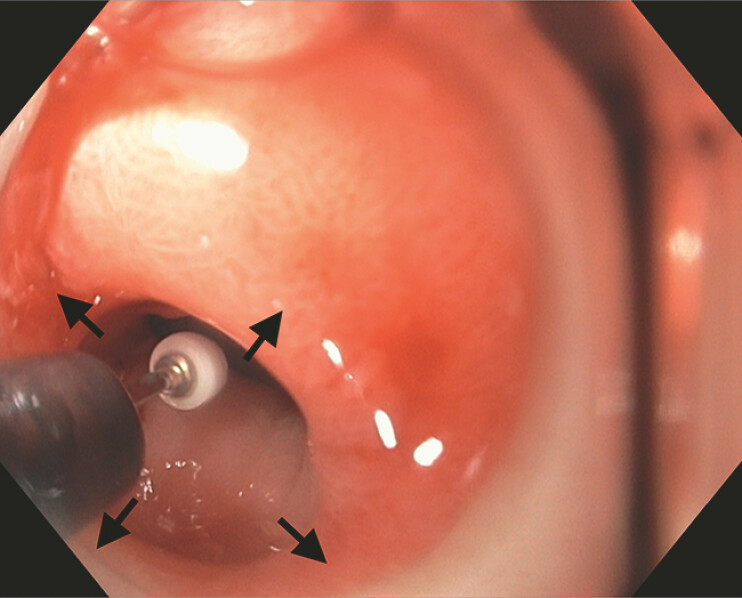
An endoscopic image depicting insulated tip nano-electrosurgical knife stricturoplasty (ITNKS) in a 3-, 6-, 9- and 12- o’clock or circumferential fashion (black arrows).

**Fig. 3:**
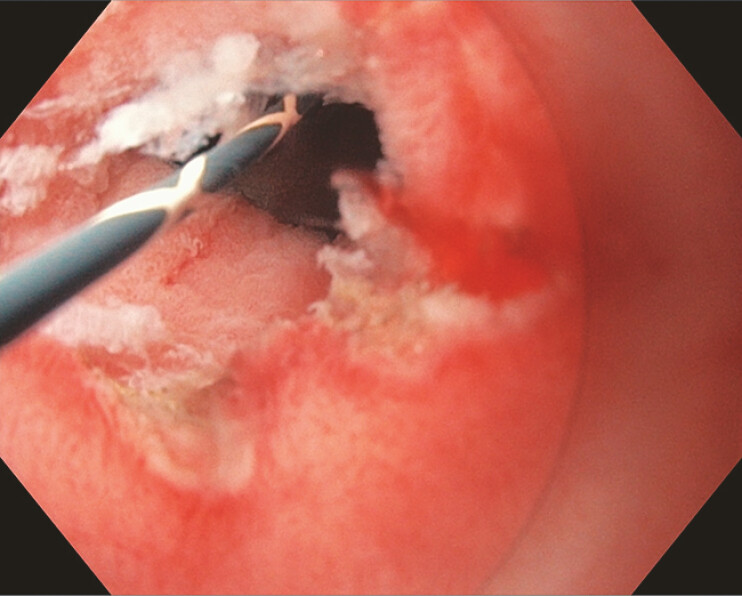
An endoscopic image of the pylorus status post-ITNKS revealing extensive underlying fibrotic tissue. ITNKS, insulated tip nano-electrosurgical knife stricturoplasty.

**Fig. 4:**
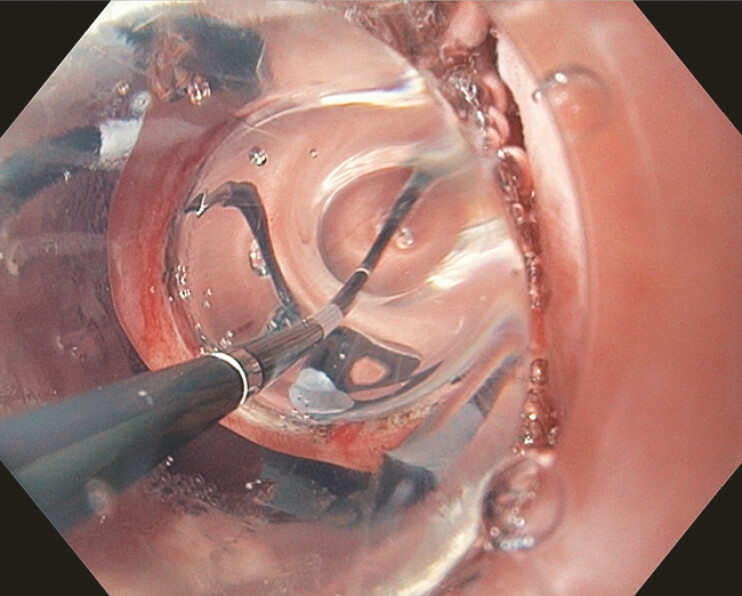
An endoscopic image depicting 13.5–15.5 mm wire-guided endoscopic balloon dilation (EBD) of the pylorus.

**Fig. 5:**
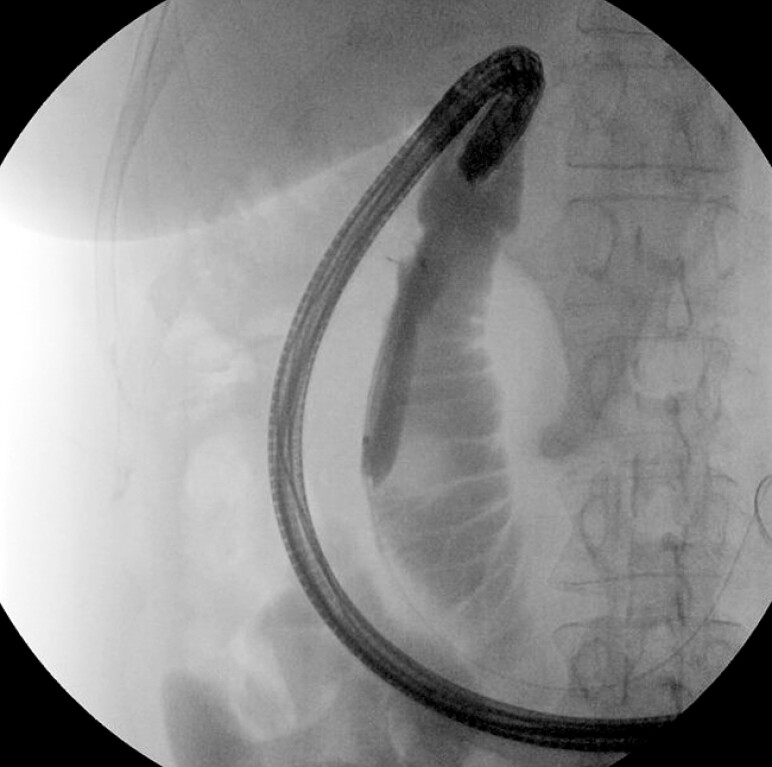
A fluoroscopic image depicting the advancement of the gastroscope into the duodenum status post ITNKS. ITNKS, insulated tip nano-electrosurgical knife stricturoplasty.

We present a case of a 67-year-old woman with a pertinent past medical history of benign, high-grade pyloric stenosis status after endoscopic balloon dilation (EBD) × 3 and an AXIOS (Boston Scientific, Marlborough, MA, USA) lumen apposing metal stent (LAMS) × 2 who presented for the endoscopic management of symptomatic, recurrent pyloric stenosis. Despite the AXIOS LAMS and EBD therapy, 22- and 7- months prior (respectively), she developed symptomatic recurrence. Esophagogastroduodenoscopy revealed a tight stricture in the distal antrum with associated pyloric stenosis ( Fig. 1 ) that precluded gastroscope passage despite 11–13 mm wire-guided EBD. She underwent endoscopic incisional therapy (EIT) via circumferential stricturoplasty ( Fig. 2 , Fig. 3 ) with an Olympus (Center Valley, PA, USA) insulated tip nano-electrosurgical knife (ITNK). Upon contrast leak absence, 13.5–15.5 mm wire-guided EBD ( Fig. 4 ) was performed which allowed for gastroscope passage ( Fig. 5 ). Intramuscular steroid injections were then performed in the four quadrants of the pylorus. One-month post-EIT, she reported resolution of her symptoms.

Initial therapy in the management of gastrointestinal strictures typically involves EBD, especially in patients with inflammatory bowel disease 1 2 and benign pyloric strictures 3 4 . Several sessions of EBD are often required to achieve luminal patency, and strictures can persist/recur despite EBD entailing alternative treatment modalities 1 3 5 . In these cases – namely benign pyloric strictures – LAMS deployment can prove beneficial as it provides sustained dilation 4 ; however, there is a risk of stricture recurrence after LAMS removal. Owing to the possibility of complications (e.g., perforation and stent migration), minimally invasive incisional endoscopic techniques such as ITNK stricturoplasty (ITNKS) have been utilized in the management of benign pyloric strictures with favorable but limited results 3 4 5 . Furthermore, its application in cases that have failed EBD and LAMS therapy are extremely scarce. As such, this case depicts the successful treatment of a pyloric stricture refractory to standard therapy via endoscopic ITNKS ( Video 1 ).

Treatment of symptomatic, recurrent pyloric stenosis refractory to endoscopic balloon dilation and lumen apposing metal stent deployment via insulated tip nano-electrosurgical knife stricturoplasty.Video 1

Endoscopy_UCTN_Code_TTT_1AO_2AH
